# Enzalutamide in patients with non-metastatic castration-resistant prostate cancer after combined androgen blockade for recurrence following radical treatment in Japan (Japanese research for patients with non-metastatic castration-resistant prostate cancer-enzalutamide: JCASTRE-zero)—a prospective single-arm interventional study

**DOI:** 10.1186/s12894-022-01096-3

**Published:** 2022-09-14

**Authors:** Mikio Sugimoto, Takuma Kato, Yoichiro Tohi, Yosuke Shimizu, Ryuji Matsumoto, Takahiro Inoue, Yutaka Takezawa, Kimihiko Masui, Hiroshi Sasaki, Hiromi Hirama, Shiro Saito, Shin Egawa, Toshiyuki Kamoto, Satoshi Teramukai, Shinsuke Kojima, Takashi Kikuchi, Yoshiyuki Kakehi

**Affiliations:** 1 Department of Urology, Faculty of Medicine, Kagawa University, 1750-1, Ikenobe, Miki-cho, Kita-gun, Kagawa 761-0793 Japan; 2 Department of Urology, Kobe City Nishi-Kobe Medical Center, Kobe, Hyogo Japan; 3 Department of Urology, Hokkaido University Graduate School of Medicine, Hokkaido, Japan; 4 Department of Urology, Graduate School of Medicine, Kyoto University, Kyoto, Japan; 5 Department of Urology, Isesaki Municipal Hospital, Isesaki, Gunma Japan; 6 Department of Urology, Otsu City Hospital, Otsu, Shiga Japan; 7 Department of Urology, Jikei University School of Medicine, Tokyo, Japan; 8 Department of Urology, National Hospital Organization Tokyo Medical Center, Tokyo, Japan; 9 Department of Urology, Faculty of Medicine, University of Miyazaki, Miyazaki, Japan; 10 Department of Biostatistics, Kyoto Prefectural University of Medicine, Kyoto, Japan; 11 Foundation for Biomedical Research and Innovation at Kobe Translational Research Center for Medical Innovation, Kobe, Hyogo Japan

**Keywords:** Combined androgen blockade, Enzalutamide, Non-metastatic castration-resistant prostate cancer, Progression-free survival, Radical treatment

## Abstract

**Background:**

The effect of enzalutamide in patients with non-metastatic castration-resistant prostate cancer after combined androgen blockade, which represents a patient profile similar to real-world clinical practice in Japan, remains unknown. Therefore, we investigate the efficacy and safety of enzalutamide after combined androgen blockade for recurrence following radical treatment in Japanese patients with non-metastatic castration-resistant prostate cancer.

**Methods:**

We analyzed 66 patients with non-metastatic castration-resistant prostate cancer after combined androgen blockade for recurrence following radical prostatectomy or radiation therapy who were prospectively enrolled from October 2015 to March 2018. They received enzalutamide 160 mg orally once daily until the protocol treatment discontinuation criteria were met. The primary endpoint was prostate-specific antigen-progression-free survival, defined as the time from enrollment to prostate-specific antigen-based progression or death from any cause. The secondary endpoints included overall survival, progression-free survival, metastasis-free survival, time to prostate-specific antigen progression, prostate-specific antigen response rate, chemotherapy-free survival, and safety assessment.

**Results:**

The median observation period was 27.3 months. The median prostate-specific antigen-progression-free survival was 35.0 months (95% confidence interval, 17.5 to not reached). The median overall survival, median progression-free survival, median metastasis-free survival, and chemotherapy-free survival were not reached, with the corresponding 2-year rates being 91.6%, 67.1%, 72.4%, and 85.8%, respectively. The 50% prostate-specific antigen response rate was 88.9%, with the median time being 2.8 months. In total, 42.2% of the patients experienced adverse events, with malaise being the most common.

**Conclusions:**

Enzalutamide effectively manages non-metastatic castration-resistant prostate cancer after combined androgen blockade for recurrence following radical treatment.

*Trial* *registration*: UMIN000018964, CRB6180007.

**Supplementary Information:**

The online version contains supplementary material available at 10.1186/s12894-022-01096-3.

## Introduction

With the widespread use of prostate-specific antigen (PSA) testing, the number of localized prostate cancer (PC) cases without metastasis are increasing in Japan; it has been reported to constitute more than 85% of PC cases [1]. The standard therapy for localized PC is radical treatment such as radical prostatectomy and radiotherapy [2]. However, unfortunately, PSA recurrence occurs within 10 years after radical treatment in 20–30% of the patients [3, 4]. PSA recurrence after radical prostatectomy is often treated with salvage radiation therapy, while subsequent recurrence is managed with hormonal therapy. Hormonal therapy is also performed for recurrence after radiation therapy; however, in both situations, the disease develops into castration-resistant PC (CRPC) within almost 5 years [5]. CRPC for which distant metastasis has not been noted on computed tomography (CT) and bone scintigraphy is termed non-metastatic CRPC (nmCRPC).

nmCRPC progressed to metastatic CRPC (mCRPC) within 1 year in 34% of cases [6]. The annual mortality rate for nmCRPC is 16% but increases to 56% for mCRPC [6]. Therefore, extending the period from nmCRPC to metastasis is crucial. Furthermore, metastases can lead to pain, fractures, and other skeletal-related events and reduce the quality of life in patients. Pain at the metastasis site may require analgesic drug treatment and palliative radiation. Therefore, the treatment of nmCRPC may have considerable economic benefits and could prolong life expectancy.

Enzalutamide, an androgen receptor axis-targeted agent (ARAT), was approved for the treatment of mCRPC before and after chemotherapy based on the PREVAIL and AFFIRM studies [7, 8]. In the placebo-controlled phase III PROSPER study in patients with nmCRPC, enzalutamide improved the median metastasis-free survival (MFS) and overall survival (OS) by 21.9 months (hazard ratio: 0.29) and 10.7 months, respectively. [9, 10] Recently, besides enzalutamide, apalutamide and darolutamide have been approved for the treatment of nmCRPC, based on the results of phase III studies [11, 12]. These studies included patients who had received radical treatment and those treated with primary androgen deprivation therapy (PADT) [9, 10, 12, 13]. Furthermore, these phase III studies included patients who received androgen deprivation therapy (ADT) alone and those who received combined androgen blockade (CAB) in combination with bicalutamide and other antiandrogen drugs [9–12]. CAB has traditionally been used as the first hormonal therapy in Japan [13]. A retrospective study of real-world use of enzalutamide in Japanese patients with nmCRPC reported the median PSA progression period and MFS to be 27 and 29 months, respectively; however, the study did not describe whether the type of PADT was ADT alone or CAB [14]. In this context, the effect of ARATs in patients with nmCRPC after CAB, which represents a patient profile similar to real-world clinical practice in Japan, remains unknown. Therefore, we evaluated enzalutamide’s effect after CAB for recurrence following radical treatment in patients with nmCRPC in the Japanese Research for Patients with Non-Metastatic Castration-Resistant Prostate Cancer – Enzalutamide: JCASTRE-Zero study.

## Methods

This study was a multi-institutional single-arm prospective trial designed in collaboration with the principal investigator and Translational Research Center for Medical Innovation, Foundation for Biomedical Research and Innovation at Kobe (TRI), and funded by Astellas Pharma, the developer of enzalutamide. It was conducted according to the Declaration of Helsinki and Clinical Trials Act, the Japanese law outlining the ethical conduct of clinical trials. The ethics review committee of Kagawa University Hospital, TRI, the individuals, and institutional review boards of all the participating facilities approved this study (trial registration: UMIN000018964, first registration: September 10, 2015). The clinical trials review board of Kagawa University Hospital also approved and certified this study in line with the provisions of the Clinical Trials Act (trial registration: CRB6180007). Informed consent was obtained from all patients. The protocol was listed at a Additional files 1 and 2.

### Participant selection

The inclusion criteria were as follows: (1) Patients aged 20 years or older with histologically confirmed PC; (2) patients with a history of radical prostatectomy or radiation therapy for radical treatment; (3) patients who received continuous ADT using either luteinizing hormone-releasing hormone (LHRH) agonist, antagonist, or surgical castration; (4) patients with a serum testosterone level of 1.73 nmol/L (0.50 ng/dL) or lower; (5) patients with a history of bicalutamide or flutamide use after the confirmation of the first recurrence since the completion of radical treatment; (6) patients with three test results showing increased PSA levels, which were measured consecutively at an interval of at least 1 week during ADT; (7) patients with a serum PSA level of 1 ng/mL or higher (2 ng/mL or more before August 23, 2017); (8) patients with no confirmed remote metastasis on CT and bone scintigraphy after the diagnosis of PC (excluding lymph node metastasis with a minor axis of less than 15 mm, which was considered non-measurable in the Response Evaluation Criteria In Solid Tumors version 1.1); (9) patients with asymptomatic PC; (10) patients with an Eastern Cooperative Oncology Group performance status of 0–1; and (11) patients with a life expectancy of at least 12 months.

The exclusion criteria were as follows: (1) patients with a history of any chemotherapy (including estramustine phosphate sodium hydrate and docetaxel) or treatment with enzalutamide or abiraterone acetate; (2) patients with a history of steroid use as a treatment for PC; (3) patients with a history of 5-alpha-reductase inhibitor, estrogen, or steroidal antiandrogen therapy within 4 weeks before the initial administration of enzalutamide; (4) patients with a history of malignant tumor other than PC within the past 3 years; (5) patients with a history of seizures or pre-disposed to seizures; (6) patients with severe liver dysfunction; and (7) patients with a history of hypersensitivity to any component of the drugs administered in this study.

### Intervention

All patients received enzalutamide 160 mg orally once daily. The treatment was started at visit 0 within 1 week after enrollment. Visit 1 occurred 2 weeks after the start of treatment; clinical assessments were conducted for adverse events (AEs) by using the Japanese version of the Functional Assessment of Cancer Therapy-Prostate (FACT-P) scales. PSA was measured every 3 months, and CT and bone scintigraphy was performed every 6 months. In the case of AEs of ≥ grade 3, enzalutamide could not be ruled out as a causative agent and was temporarily discontinued. However, after the AE severity reduced to grade 1 or less, enzalutamide was resumed at half the original dose (80 mg) initially. Eligible patients in this study continued the treatment until the 12th-week visit (counted from the administration of the initial dose) until they met the withdrawal or protocol treatment discontinuation criteria. The outcomes for these patients were evaluated for 2 years from the final patient enrollment.

### Endpoints

The primary endpoint was PSA-progression-free survival (PSA-PFS), defined as the time from enrollment to PSA-based progression or death from any cause. PSA-based progression was defined as an increase in PSA levels of > 25% relative to the nadir PSA level and higher than 2 ng/mL [15].

The secondary endpoints included OS, defined as the time from enrollment to death from any cause, PFS, and MFS. PFS was defined as the time from enrollment to investigator-assessed disease progression (based on imaging or symptom development) or death from any cause. MFS was defined as the time from enrollment to disease progression assessed by CT and bone scintigraphy by investigator review. Other secondary endpoints were a 50% PSA response rate (less than half the baseline PSA concentration), chemotherapy-free survival, and quality of life (QOL) assessment using the Japanese version of the FACT-P scales. A safety assessment of the frequency and severity of AEs was performed using the Common Terminology Criteria for Adverse Events (CTCAE) version 4.0 [16].

### Data collection

Information on patients’ demographic and disease characteristics; interventions; laboratory tests; and outcome evaluations, including PSA, imaging, and survival, were obtained by investigators at each participating institute via a filled-in form on the website prepared by TRI. The PSA response rates at 2 and 12 weeks after the start of treatment were reported as the number and percent of patients with 95% confidence intervals (CI). The health-related QOL (assessed using the FACT-P scales) data at baseline and at 2 and 60 weeks after the start of treatment were collected.

### Statistical analysis

We carried out univariate analyses for time-to-event data in primary and secondary endpoints by using the Kaplan–Meier method; the median survival time and 95% CI were estimated. We also summarized patient characteristics with frequencies and percentages for categorical variables or with medians and ranges for continuous variables.

As an ad-hoc analysis, we investigated the PSA concentrations in the follow-up period as potential predictors of OS or PFS using a Cox proportional hazard model with PSA as a time-dependent variate and baseline age as a covariate. Furthermore, we performed a restricted cubic spline regression (RCSR) with three knots, assuming the Cox proportional hazard ratio could be nonlinearly changed according to the changes in PSA concentrations [17]. This model evaluated PSA concentrations not only at baseline but also at other each patient’s visit until the patient experienced the outcome event or was censored. The aim of this approach was to test the following null hypothesis: PSA concentration curves over time were identical between censored and event-experienced patients. If the null hypothesis is rejected, it would indicate that event-experienced patients had characteristic changes in PSA concentrations over time, which were different from those of censored or survived patients. We used SAS statistical software (version 9.3, SAS Institute Inc., Cary, NC, USA), R4.2.1 (R Foundation), and R library *rms* for the RCSR.

### Sample size determination

The sample size was determined to be 60 evaluable subjects for the feasibility of this study. However, with a null hypothesis that states the practical lowest effective limit of the median PSA-PFS time was 13 months and considering that the intervention could achieve 24 months of the median PSA-PFS time, the sample size could reject the null hypothesis with a power of 0.87 and 5% significance level in a two-sided test.

## Results

### Patients

From October 1, 2015, to March 30, 2018, 66 patients were enrolled. The first patient was registered in January 13, 2016 (Number: 020–001). After the enrollment, two patients were not treated with enzalutamide because of a registration error and withdrawal of consent. We, therefore, evaluated 64 patients as a safety analysis dataset (SAS).

The mean patient age (standard deviation) was 75.4 years (6.5 years), and the median PSA level was 3.95 ng/mL in the SAS. Other patient characteristics are shown in Table 1. The median observation period was 24.7 months (95% CI 24.4–35.4 months). All 64 patients received enzalutamide for two weeks. Moreover, 42, 16, and 5 patients received enzalutamide over 60, 120, and 180 weeks, respectively. The median treatment duration was 24.7 months (95% CI 0.5–50.2 months).

**Table 1:** Patients characteristics

			N = 64	
Age	years			
Mean (SD)			75.4	6.5
Median			76	
Range			58–90	
ECOG performance-status	No. (%)			
0			63	98.4
1			1	1.6
TNM classification (cT)	No. (%)	T1a	1	1.6
		T1c	7	10.9
		T2a	10	15.6
		T2b	5	7.8
		T2c	9	14.1
		T3a	19	29.7
		T3b	9	14.1
		T4	2	3.1
		TX	2	3.1
TNM classification (cN)	No. (%)	N0	60	93.8
		N1	3	4.7
		NX	1	1.6
Gleason score	No. (%)	≤ 6	8	12.5
		7	18	28.1
		8 ≤	38	59.4
Serum testosterone	ng/dL			
Mean (SD)			11.85	11.37
Median			10	
Range			0.03–47	
Serum PSA	ng/mL			
Mean (SD)			6.7	7.66
Median			3.95	
Range			1.03–35.13	
History of radical prostatectomy			29	45.3
With endocrine therapy			14	48.3
TNM classification (pT)	T1c		1	3.4
	T2a		3	10.3
	T2b		4	13.8
	T2c		3	10.3
	T3a		6	20.7
	T3b		8	27.6
	T4		2	6.9
	TX		2	6.9
TNM classification (pN)	N0		24	82.8
	N1		3	10.3
	NX		2	6.9
Gleason score	≤ 6		1	3.4
	7		8	27.6
	8 ≤		19	65.5
	Missing		1	3.4
History of radiation therapy	Yes		48	75.0
	EBRT		43	89.6
	Brachytherapy		4	8.3
	EBRT + Brachytherapy		1	2.1
	with endocrine therapy		27	56.3
History of bilateral orchiectomy			1	1.6
History of GnRH analogue use			63	98.4
History of bicalutamide use			64	100
Median administration period	day		715	
AWS	yes		46	71.9
History of flutamide use			23	35.9
Median administration period	day		330	
AWS	yes		16	25.0

*AWS* Antiandrogen withdrawal syndromes; *cN* Clinical lymph nodes classification; *cT* Clinical tumor classification; *EBRT* External beam radiotherapy; *ECOG* Eastern cooperative oncology group; *GnRH* Gonadotropin-releasing hormone; *pN* Pathological lymph node classification; *PSA* Prostate-specific antigen; *pT* Pathological tumor classification; *SD* Standard deviation

Nine patients were excluded from the SAS due to violation of eligibility or dose-reduction criteria. Thus, there were 55 patients in the full analysis dataset (FAS), and the clinical effects were evaluated.

### Primary endpoint: PSA-PFS

**Fig. 1:**
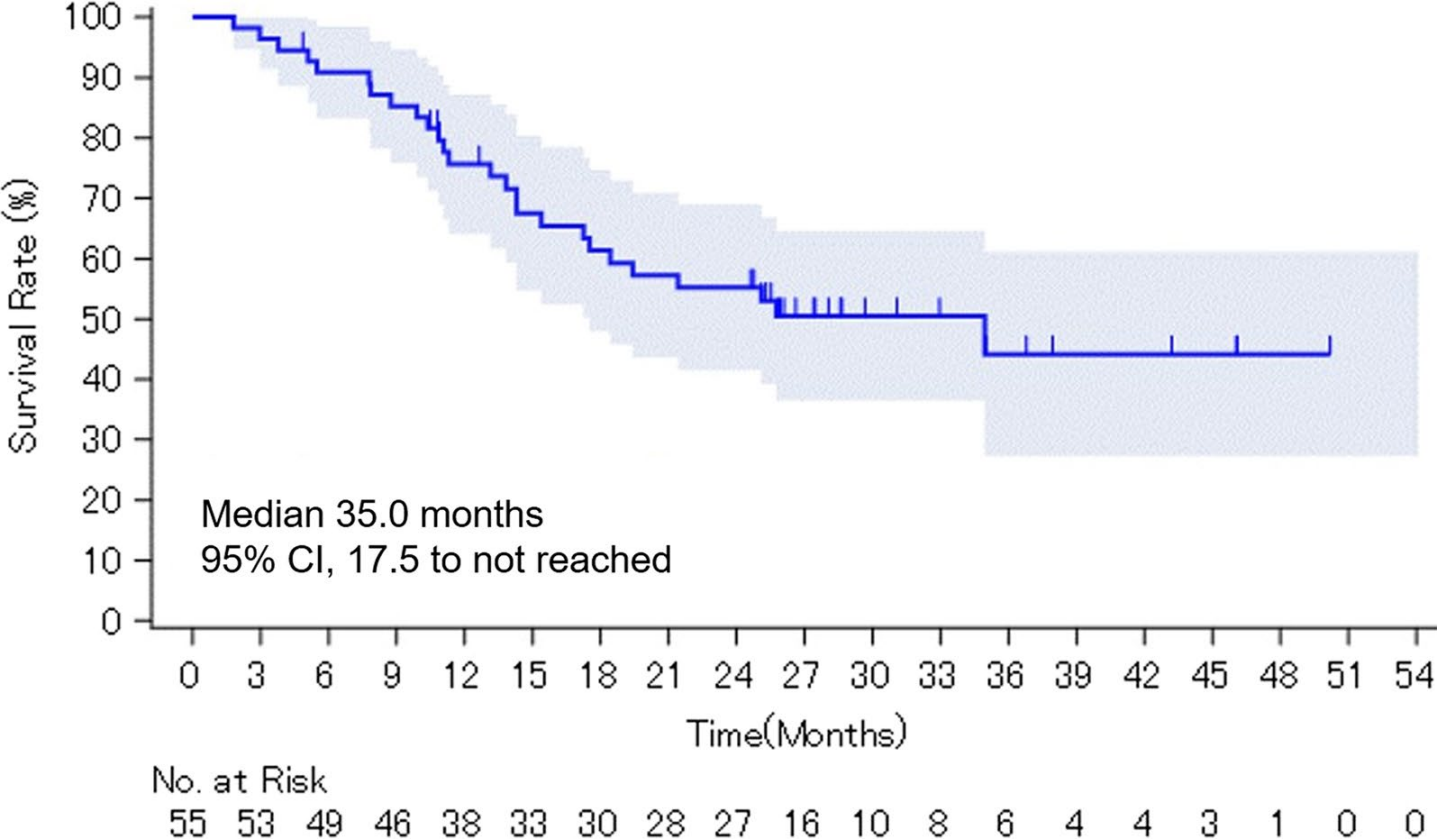
Kaplan–Meier method for PSA-progression-free survival estimation, PSA, prostate-specific antigen; CI, confidence interval.

Twenty-six subjects (47%) experienced PSA-progression or death. The median PSA-PFS time was 35.0 months (95% CI 17.5 to not reached; Fig. 1). The estimated PSA-PFS rates at 12, 24, and 36 months were 77.1%, 56.2%, and 45%, respectively.

### Secondary endpoints

**Fig. 2:**
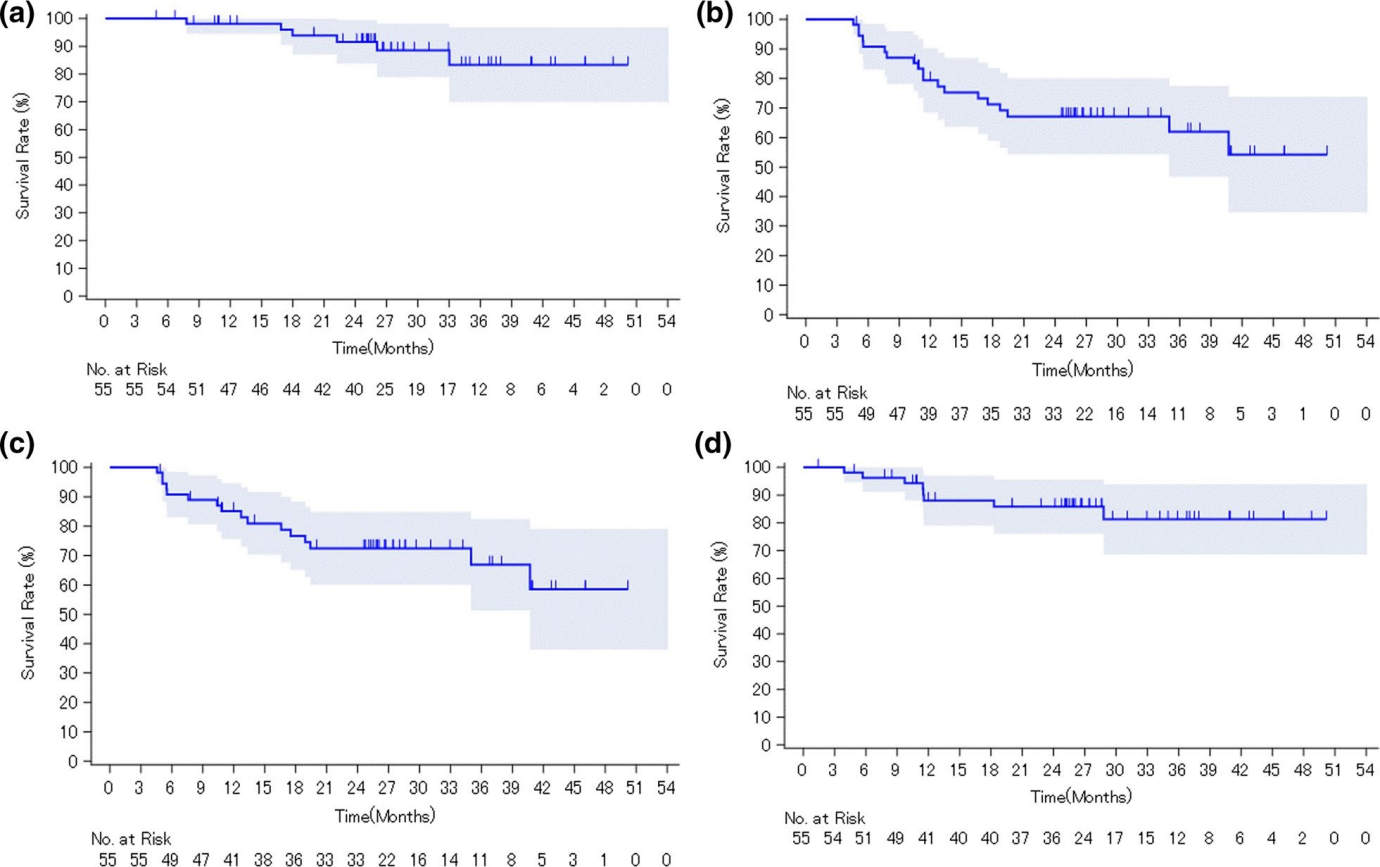
Kaplan–Meier method for the estimation of **a** overall survival, **b** progression-free survival, **c** metastasis-free survival, and **d** chemotherapy-free survival. *PSA* Prostate-specific antigen; *CI* confidence interval

The median OS, PFS, MFS, and chemotherapy-free survival were not reached (Fig. 2a, b, c, and d). The 2-year OS, PFS, MFS, and chemotherapy-free survival rates were 91.6%, 67.1%, 72.4%, and 85.8%, respectively. The 50% PSA response rate was 88.9%, with the median time being 2.8 months (95% CI 2.8–3.1 months).

Regarding the HRQOL (FACT-P) analysis, the mean total scores 2 and 60 weeks after treatment initiation were not significantly different from those at the study initiation time (Additional file 1: Figure).

### Safety

Adverse effects of any grade were found in 27 patients (42.2%), and those of grade ≥ 3 were found in 16 patients (25.0%). The AEs are summarized in the Additional file 2: Table. The most frequently reported AE of any grade and grade ≥ 3 was malaise (10 patients [15.6%] and 3 patients [4.7%], respectively).

### Ad-hoc analysis: PSA concentration as a time-dependent covariate in the OS and PFS analyses

**Fig. 3:**
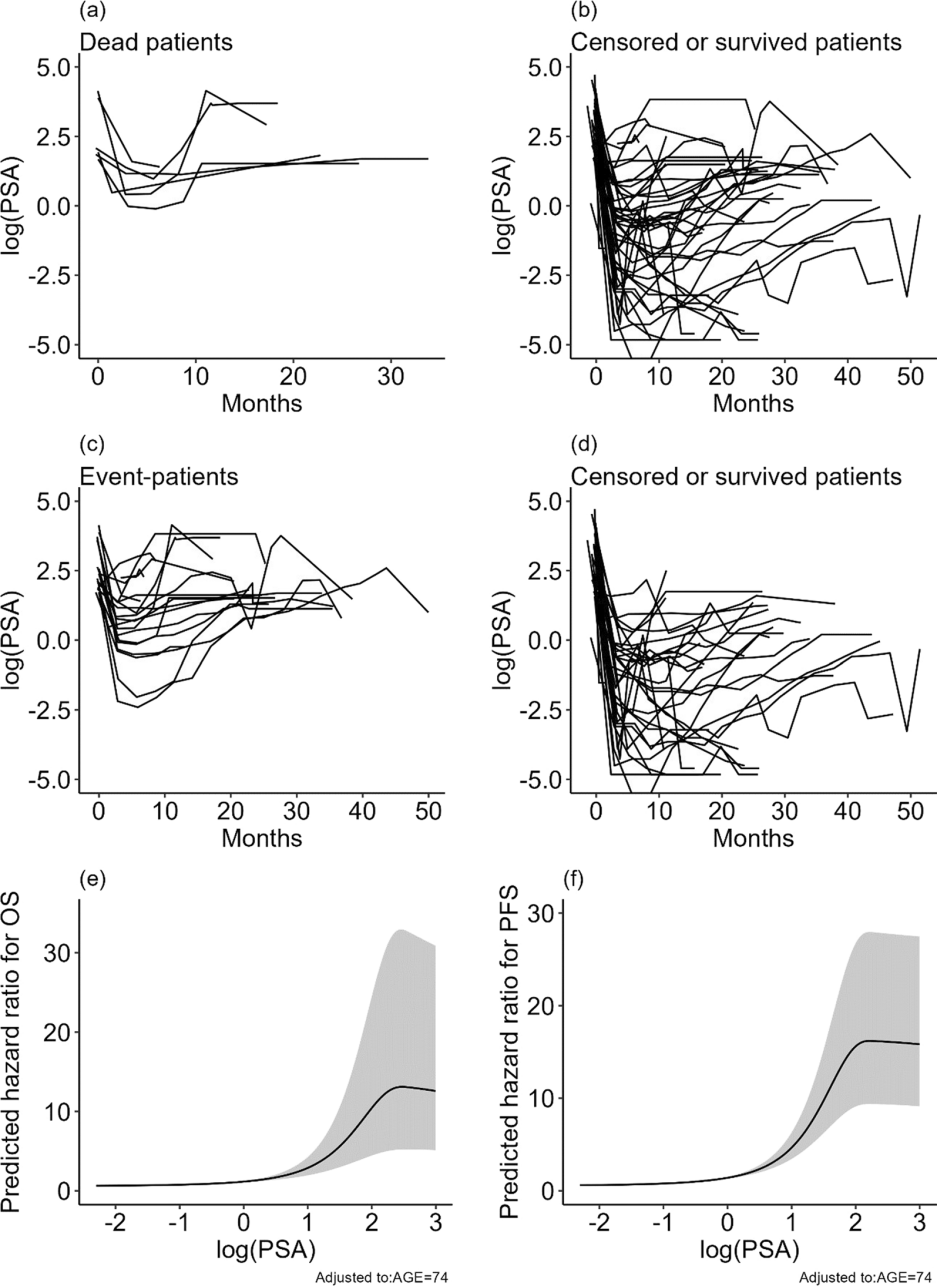
Relationship between PSA concentrations during the therapy and OS or PFS. Time-series changes in log (PSA) for dead patients in OS (**a**) and censored or surviving patients (**b**); Time-series changes in log(PSA) for deceased patients in PFS (**c**) and censored or survived patients (d); changes in a predicted hazard ratio by log(PSA) for OS (**e**) and PFS (**d**) with adjusted AGE to 74, the median age of the patients. The PSA concentrations in the dead (**a**) and progressed patients (**c**) failed to decrease sufficiently, and the concentrations increased before ten months. The behaviors of PSA concentration curves were different between event-patients and survived or censored patients. Simulated changes in OS risk (**e**) and PFS risk (**f**) for a model case (baseline age 74) show that if the log(PSA) > 1 or PSA > 2.7 ng/mL, then the risk can rapidly increase.

Figure 3 shows the time-series changes in PSA concentrations in logarithms for patient events, censored or surviving patients, and the predicted hazard ratio for OS and PFS by the PSA concentrations with RCSR.

The mean linear effect on the hazard ratio for OS by PSA concentrations in the spline function (95% confidence interval; *p*-value) was 1.98 (1.48–2.65; < 0.001). Since the effect is larger than 1.0, the hazard tended to increase nonlinearly as PSA concentration increased during the treatment period. In contrast, when we evaluated PSA concentration by controlling age at baseline, the PSA concentrations was not significant (*p*-value = 0.413).

Similarly, the mean linear effect on the hazard ratio for PFS (95% confidence interval; *p*-value) by PSA concentrations was 2.72 (2.07–3.56; < 0.001). Again, the hazard increased as PSA concentration increased during the treatment period. PSA concentrations by controlling age at baseline did not show significance for PFS (*p*-value = 0.122), either.

## Discussion

Our study aimed to assess the efficacy and safety of enzalutamide after CAB for recurrence following radical treatment in Japanese patients with nmCRPC. JCASTRE-Zero was a single-arm study where PSA-PFS longer than 24 months was achieved (35.0 months), which was the lower limit of the expected median survival time at the time of study planning. The median OS, PFS, MFS, and chemotherapy-free survival were not reached. HRQOL (FACT-P) analysis did not reveal any significant change in QOL. We believed that the efficacy of enzalutamide was highly relevant in patients with nmCRPC after CAB, which represents a patient profile similar to that in real-world clinical practice in Japan. Therefore, our study findings are meaningful for providers in real-world clinical practice in the context of patients’ treatment decision making and counseling.

The median time to PSA progression was 37.2 months in the PROSPER study [9] and 35.0 months in our study. Further, the median OS was 67.0 months in the PROSPER study [10]. As the median OS was not reached in our study, it could not be compared to the PROSPER study. However, from the PROSPER study, the 36-month survival rate was estimated to be approximately 85%, [10], which was comparable to that in our study (91.6%). The median MFS was 36.6 months in the PROSPER study. However, in our study, the median MFS was not reached, and the MFS rate at 36 months was 67%. Moreover, the median time to chemotherapy was 36.6 months in the PROSPER study, and the CFS rate at 36 months was 85.8% in our study. Although the results of both studies were similar in terms of the median PSA-PFS and OS rate, interestingly, the changes in the survival curves of MFS and CFS were more gradual in our study. This discrepancy may be due to the difference in the background of patients with nmCRPC between the JCASTRE-Zero and PROSPER studies.

In recent years, apalutamide and darolutamide, as well as enzalutamide, were approved in the nmCRPC treatment space. Their phase III studies showed that the median MFS was 40.5 months in the SPARTAN study and 40.4 months in the ARAMIS study, respectively [11, 12]. In our study, because the median MFS were not reached and the MFS rate at 36 months was 67%, our result is comparable with other ARAT studies for nmCRPC.

Of note, the baseline PSA was lower in our study than that in the PROSPER study. The median baseline PSA was 3.95 ng/mL in our study and 11.1 ng/mL in the PROSPER study [9]. The difference in the baseline PSA between the two studies may be attributed to a small tumor volume after radical treatment in all patients in our study and an inclusion criterion of PSA greater than 1.0 ng/mL. Furthermore, our study had no restriction regarding the PSA-doubling time at enrollment. These differences in baseline characteristics may have led to differences in outcomes between the JCASTRE-Zero and PROSPER studies.

Our study's safety profile of enzalutamide was consistent with that reported in previous clinical trials involving patients with CRPC [7–10]. The incidence of AEs of grade 3 or higher was 17.0 per 100 person-years in the PROSPER study [9] and 13.5 per 100 person-years in our study. Malaise was the most common AE in (15.6%, 10/64 patients) in our study; however, this incidence was lower than that in the PROSPER study (46%) [10]. In addtion, in our study, HRQOL did not worsen during the treatment with enzalutamide. This means that enzalutamide might have a clinical benefit in terms of maintaining QOL for the patients with nmCRPC.

Many papers discussed the risk of OS or PFS based on the PSA concentration at baseline. However, to the best of our knowledge, studies statistically investigating the impact of PSA concentration on OS risk or PFS during treatment have not been reported. To analyze this relationship, we conducted an ad-hoc study that included PSA concentration at each visit as a time-dependent variable in a Cox proportional model [17]. We found a positive relationship with a significant OS risk and PFS increases with increasing PSA concentrations. However, no correlation was observed between the baseline PSA concentrations and OS or PFS. As shown in Fig. 3, the PSA concentration for patients who experienced the events tended to be higher than that for censored or surviving patients during the study period. The predicted hazard ratios in OS and PFS were drastically increased beyond log(PSA) = 1 or PSA = 2.72 ng/mL. This result suggested that monitoring PSA concentration is essential to managing the risk in OS and PFS.

Our study has several limitations. First, since this study was not a controlled study, the effectiveness of enzalutamide in patients with nmCRPC could not be directly discussed. Differences in various baseline factors made simple comparisons with the PROSPER study difficult. In other words, our study had different baseline PSA because all patients received radical treatment. Second, as JCASTRE-Zero was planned before the results of the PROSPER study were published, we assumed that using the same inclusion criteria as the PROSPER study would not be novel enough. Therefore, JCASTRE-Zero was set to include patients who underwent CAB for recurrence after radical treatment. Furthermore, PSA-DT was not set as the inclusion criterion in JCASTRE-Zero because we considered it a disadvantage in terms of case collection. Therefore, PSA-DT was not available. However, nmCRPC after CAB is a disease status that reflects a real-world clinical issue in Japan, and our data are highly relevant to day-to-day clinical practice.

## Conclusion

In Japanese patients with nmCRPC after CAB following radical treatment, enzalutamide treatment resulted in a comparable PSA-PFS and a longer MFS compared to those in the PROSPER study. Enzalutamide treatment can be an option for nmCRPC patients.

## Supplementary Information

**Additional** **file 1.** **Supplementary figure.** Change in the functional assessment of cancer therapy-prostate total score.

**Additional** **file 2.** **Supplementary table.** Summary of adverse events.

## Data Availability

The datasets used and analysed in the current study are from the corresponding author on reasonable request.

## Abbreviations

PSA: Prostate-specific antigen
PC: Prostate cancer
CRPC: Castration-resistant PC
CT: Computed tomography
nmCRPC: Non-metastatic CRPC
mCRPC: Metastatic CRPC
ARAT: Androgen receptor axis-targeted agent
PADT: Primary androgen deprivation therapy
MFS: Metastasis-free survival
OS: Overall survival
ADT: Androgen deprivation therapy
CAB: Combined androgen blockade
FACT-P: Functional assessment of cancer therapy-prostate
PSA-PFS: PSA-progression-free survival
QOL: Quality of life
RCSR: Restricted cubic spline regression
