# Supplementary figures and images for: Enzalutamide in patients with non-metastatic castration-resistant prostate cancer after combined androgen blockade for recurrence following radical treatment in Japan (Japanese research for patients with non-metastatic castration-resistant prostate cancer-enzalutamide: JCASTRE-zero)—a prospective single-arm interventional study
Source: BMC Urol. 2022 Sep 14;22:151. doi: 10.1186/s12894-022-01096-3 (PMC9476281; doi:10.1186/s12894-022-01096-3)

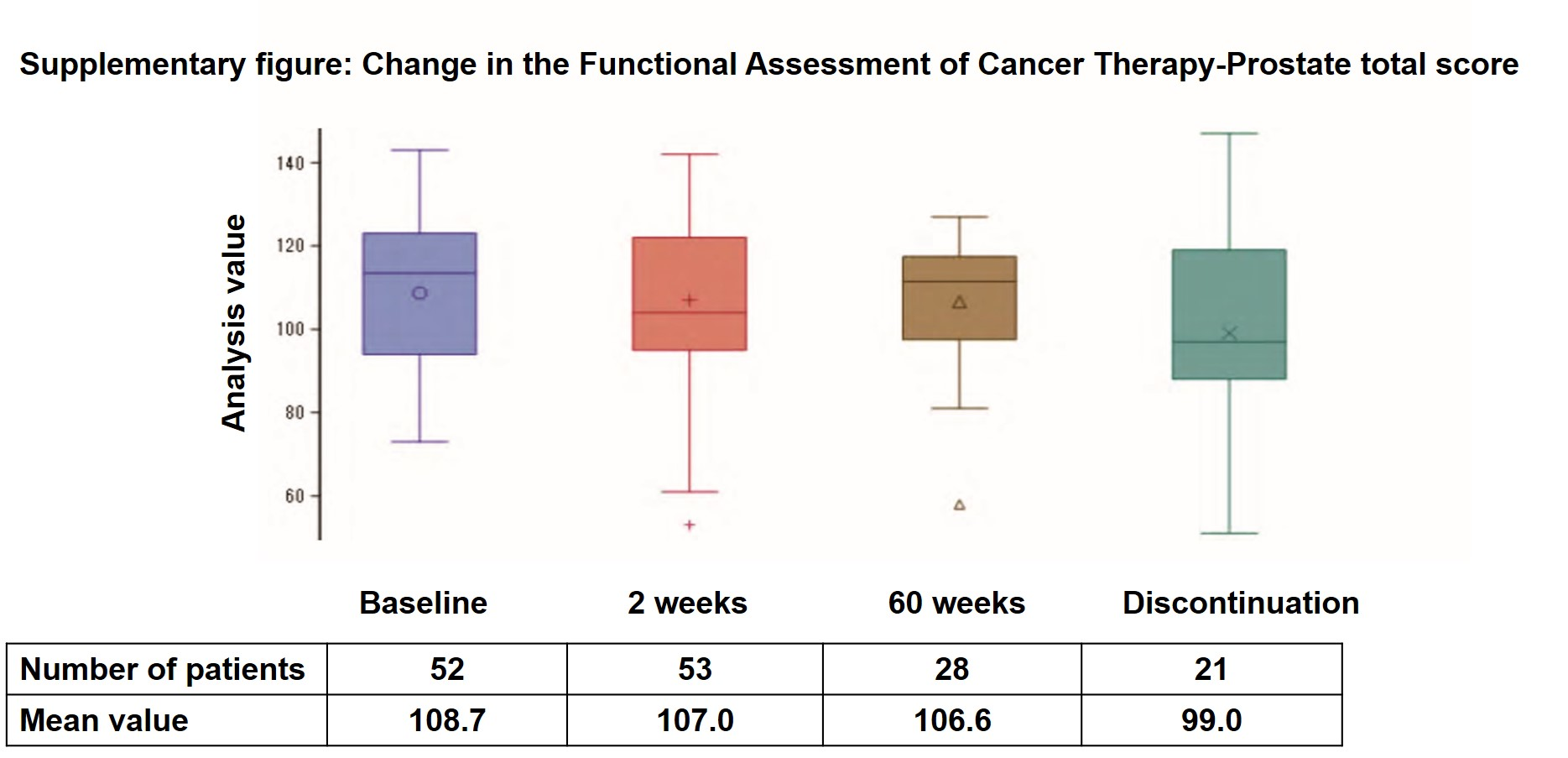

Supplement: Supplementary file 1 — Additional file 1. Supplementary figure. Change in the functional assessment of cancer therapy-prostate total score. [file 12894_2022_1096_MOESM1_ESM.tif]
