# Supplementary material for: Enzalutamide in patients with non-metastatic castration-resistant prostate cancer after combined androgen blockade for recurrence following radical treatment in Japan (Japanese research for patients with non-metastatic castration-resistant prostate cancer-enzalutamide: JCASTRE-zero)—a prospective single-arm interventional study
Source: BMC Urol. 2022 Sep 14;22:151. doi: 10.1186/s12894-022-01096-3 (PMC9476281; doi:10.1186/s12894-022-01096-3)
Supplement: Supplementary file 2 — Additional file 2. Supplementary table. Summary of adverse events. [file 12894_2022_1096_MOESM2_ESM.docx]

Supplementary table Summary of Adverse Events

| Name of AE | | Total | | ≥Grade 3 | |
| --- | --- | --- | --- | --- | --- |
|  |  | No. | % | No. | % |
| Blood and lymphatic system disorders | | 1 | 1.6 | 1 | 1.6 |
|  | Neutropenia | 1 | 1.6 | 1 | 1.6 |
| Cardiac disorders | | 1 | 1.6 | 1 | 1.6 |
|  | Myocardial infarction | 1 | 1.6 | 1 | 1.6 |
| Ear and labyrinth disorders | | 1 | 1.6 |  |  |
|  | Vertigo | 1 | 1.6 |  |  |
| Gastrointestinal disorders | | 6 | 9.4 | 1 | 1.6 |
|  | Constipation | 2 | 3.1 |  |  |
|  | Diarrhea | 2 | 3.1 |  |  |
|  | Intestinal obstruction | 1 | 1.6 | 1 | 1.6 |
|  | Nausea | 3 | 4.7 |  |  |
| General disorders and administration site conditions | | 10 | 15.6 | 3 | 4.7 |
|  | Malaise | 10 | 15.6 | 3 | 4.7 |
| Hepatobiliary disorders | | 2 | 3.1 |  |  |
|  | Liver dysfunction | 2 | 3.1 |  |  |
| Infections and infestations | | 1 | 1.6 | 1 | 1.6 |
|  | Urinary tract infection | 1 | 1.6 | 1 | 1.6 |
| Investigations | | 4 | 6.3 | 2 | 3.1 |
|  | Neutrophil count decreased | 1 | 1.6 | 1 | 1.6 |
|  | Platelet count decreased | 2 | 3.1 | 1 | 1.6 |
|  | Weight loss | 1 | 1.6 |  |  |
| Metabolism and nutrition disorders | | 8 | 12.5 | 2 | 3.1 |
|  | Glucose intolerance | 1 | 1.6 | 1 | 1.6 |
|  | Anorexia | 8 | 12.5 | 1 | 1.6 |
| Neoplasms benign, malignant and unspecified (incl cysts and polyps) | | 2 | 3.1 | 2 | 3.1 |
|  | Gastric cancer | 1 | 1.6 | 1 | 1.6 |
|  | Hypopharyngeal cancer | 1 | 1.6 | 1 | 1.6 |
| Nervous system disorders | | 2 | 3.1 | 1 | 1.6 |
|  | Cerebral hemorrhage | 1 | 1.6 | 1 | 1.6 |
|  | Dizziness | 1 | 1.6 |  |  |
|  | Dysgeusia | 1 | 1.6 |  |  |
| Skin and subcutaneous tissue disorders | | 3 | 4.7 | 2 | 3.1 |
|  | Drug eruption | 2 | 3.1 | 2 | 3.1 |
|  | Rash | 1 | 1.6 |  |  |
| Surgical and medical procedures | | 1 | 1.6 | 1 | 1.6 |
|  | Prevention of gastrointestinal disorders | 1 | 1.6 | 1 | 1.6 |
| Vascular disorders | | 2 | 3.1 | 1 | 1.6 |
|  | Hypertension | 2 | 3.1 | 1 | 1.6 |

AE, Adverse Events
